# Hospital admission for hyperemesis gravidarum in women at increased risk of spontaneous preterm birth

**DOI:** 10.1111/birt.12303

**Published:** 2017-08-30

**Authors:** Ira Kleine, Ana Da Silva, Wafaa Ahmed, Frida Forya, Sara M. Whitten, Anna L. David, Catherine P. James

**Affiliations:** ^1^ UCL Medical School London UK; ^2^ Fetal Medicine Unit University College London Hospitals NHS Foundation Trust London UK; ^3^ UCL Institute for Women's Health London UK

**Keywords:** early pregnancy admissions, hyperemesis gravidarum, preterm birth

## Abstract

**Background:**

Progesterone administration prevents spontaneous preterm birth (sPTB) in women at increased risk**.** Progesterone concentration is lower in women with subsequent sPTB. Conversely, high concentrations of progesterone are implicated in the pathogenesis of hyperemesis gravidarum (HG). We hypothesized that women at increased risk of sPTB or spontaneous late miscarriage would be less likely to have a diagnosis of HG. To explore this hypothesis, we compared the incidence of HG in women at increased risk of sPTB and women with no identifiable risk factors.

**Methods:**

Women at increased risk of sPTB were identified from a specialist Preterm Birth Clinic (PTBC) database where criteria for PTBC attendance are previous cervical surgery, previous sPTB <34 weeks, previous spontaneous late miscarriage, incidental sonographic cervical shortening, and uterine anomaly. Hospital antenatal booking and coding records for the same time period were examined to identify HG admissions. Women with multiple gestations, trophoblastic disease, or pre‐existing abnormal thyroid function were excluded. The incidence of HG among PTBC (n=394) and non‐PTBC attendees (n=4762) was calculated.

**Results:**

The incidence of HG was lower in women at increased risk of sPTB (1.52%, n=6) compared with women with no identifiable risk factor for sPTB (3.33%, n=159; *P*=.049).

**Conclusion:**

Hospital admission for HG is reduced in women with risk factors for sPTB compared with those without risk factors. Exploration of the pathogenesis of HG may improve understanding of the mechanisms underlying sPTB.

## 1 BACKGROUND

Altered progesterone concentration is implicated in the pathogenesis of both hyperemesis gravidarum (HG) and spontaneous preterm birth (sPTB). Antenatal administration of progesterone prevents sPTB in women at increased risk.^1^ Low concentration of salivary progesterone was found to be associated with recurrent sPTB in a retrospective cohort of women at increased risk of sPTB,^2^ an observation that has been supported by more recent prospectively collected data.^3^, ^4^, ^5^ Historically HG is more common in multiple pregnancy where progesterone concentrations are higher. A recent meta‐analysis^6^ identified two studies showing an association between high serum progesterone concentration and HG.^7^, ^8^ It seems plausible, therefore, that HG and sPTB are the clinical manifestations of opposite extremes of a biochemical spectrum, although the complexity of the fetal‐placental unit makes identifying a single factor and its role challenging.

Studies investigating the relationship between HG and sPTB have thus far been contradictory; some indicate that HG is a risk factor for sPTB,^9^, ^10^ while others state that there is either no effect,^11^ or that a diagnosis of HG may be protective.^12^ Stratification for risk of sPTB generally uses the past obstetric and surgical history. In particular, women who have experienced at least one sPTB or spontaneous late miscarriage are known to be at increased risk of recurrent sPTB.^13^ Other risk factors for sPTB and spontaneous late miscarriage include: cervical surgery such as large loop excision of the transformation zone, or cone biopsy^14^; uterine anomaly^15^; incidental finding of a short cervical length^16^; and uterine surgery.^17^

We hypothesized that women at increased risk of sPTB or spontaneous late miscarriage would be less likely to have a diagnosis of HG. While the term HG is used relatively loosely in clinical practice, the NICE guidance defines HG as “prolonged and severe nausea and vomiting, dehydration, electrolyte imbalance, ketosis, the need for admission to hospital, and body weight loss of more than 5% of pre‐pregnancy weight.”^18^ We examined our hypothesis by comparing the incidence of HG as defined above in women at increased risk of sPTB with those women with no risk factors for sPTB identifiable at booking.

## 2 METHODS

**Figure 1:**
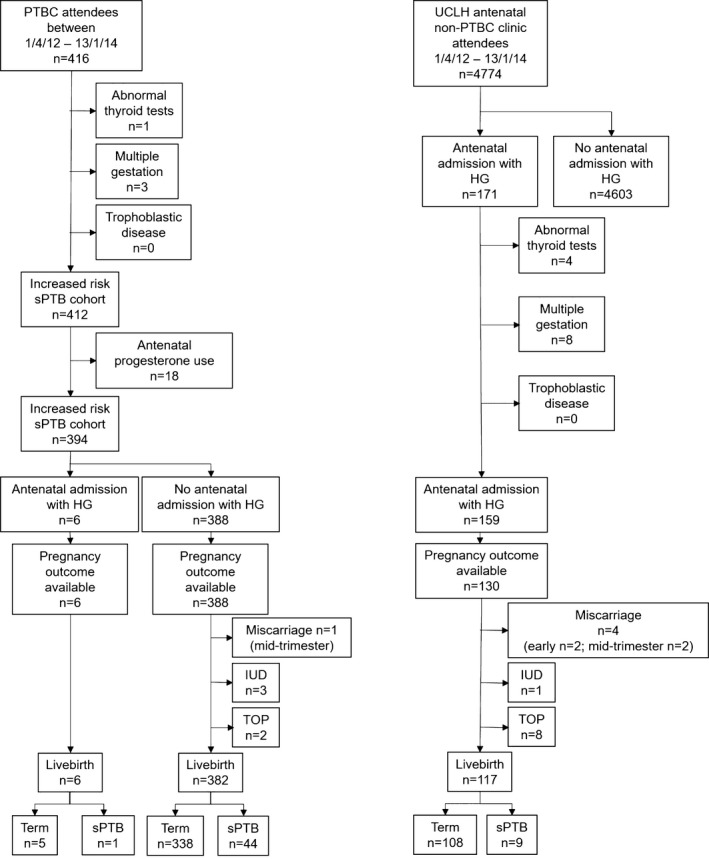
Case identification process and pregnancy outcomes in women at increased risk of sPTB (PTBC cohort) and those not at increased risk (non‐PTBC cohort) in a retrospective cohort study at UCLH between April 2012 and January 2014. Records were identified by the PTBC database and the Medical Coding and Information Systems departments; the final groups were defined after applying the exclusion criteria. The overall sPTB rate for women attending the PTBC was 11.4% (n=45). Pregnancy outcome data were available for 81.8% (n=130) of women not attending the PTBC; of this group the overall sPTB rate was 7.7%. IUD, intrauterine death; TOP, termination of pregnancy

Electronic records for all women booking at University College London Hospital (UCLH) between April 2012 and January 2014 were identified by the Medical Coding and Information Systems departments. Women booking in this period who were referred to the Preterm Birth Clinic (PTBC) were identified from the prospectively collected PTBC database; Table 1 describes the referral criteria. These women were subsequently considered separately from the total antenatal booking population, thus creating two groups: (1) women booking between April 2012 and January 2014 identified as being at increased risk of sPTB, n=394 (PTBC cohort), and (2) women booking in the same time period with no identifiable risk factors, n=4762 (non‐PTBC cohort). Women with multiple gestations, trophoblastic disease, and abnormal thyroid function were excluded.^19^ We examined the casenotes and PTBC database for evidence of progesterone treatment in the first and second trimesters; these women were subsequently excluded (Figure 1).

**Table 1:** Reasons for referral to the PTBC among the cohort of women seen in PTBC at UCLH between April 2012 and January 2014

Large Loop Excision of the Transformation Zone (LLETZ)	165
Cervical cone biopsy	46
Previous sPTB	87
Previous spontaneous mid‐trimester/late miscarriage	51
Incidental finding of a short cervical length	12
Uterine anomaly	25
Ehlers Danlos Syndrome/hypermobility	6
Uterine surgery	2
Total	394

Hospital admissions for HG were retrieved from the Medical Coding using ICD‐10 codes O21.0 (mild HG) and O21.1 (HG with metabolic disturbance); n=6 for women attending the PTBC and n=159 for the women with no risk factor for sPTB. Figure 1 shows a diagrammatic representation of the inclusion process.

VassarStats (http://vassarstats.net/) was used to calculate the significance of the difference between the proportions of women with HG in the two groups (Table 2). The need for institution ethical review board approval was waived by the NHS Health Research Authority which deemed that the study was not research, since this was analysis of routinely collected local hospital patient data.

## 3 RESULTS

Eighteen women who attended the PTBC during the study period were treated with progesterone (median gestational age: 12 weeks [2 + 0 to 20 + 5 weeks]; gestational data unavailable for three women); none were admitted with HG. Fourteen of the 394 women included in the study took part in the OPPTIMUM randomized control trial, which investigates the use of progesterone in preventing sPTB, taking either progesterone or placebo after 20 weeks of gestation^20^; none of these women were admitted with HG, although the timing of this progesterone use anyhow precludes it from contributing to first‐trimester HG presentations. Forty out of 416 women who attended the PTBC had a cervical cerclage procedure; none were admitted with HG. Treatment with progesterone and/or cerclage is not offered to non‐PTBC women.

Six out of 412 women (1.46%) in the PTBC cohort were admitted with HG, compared with 159 out of 4762 women (3.33%) in the non‐PTBC cohort (z ratio 2.086, *P* = .037). When excluding women treated with progesterone from analysis, the difference in proportions remained significant (6 of the remaining 394 PTBC cohort women [1.52%] were admitted with HG; z = 1.97, *P* = .049; Table 2).

**Table 2:** Incidence of HG in the women at increased risk of sPTB (PTBC cohort) and those not at increased risk (non‐PTBC cohort) in a retrospective cohort study at UCLH between April 2012 and January 2014

Incidence of HG in PTBC cohort	Incidence of HG in non‐PTBC cohort	*P* value (*z* ratio)
1.52% (6/394)	3.33% (159/4762)	0.049 (1.97)

## 4 DISCUSSION

Among the antenatal population of UCLH, we observed that the incidence of HG, as defined by ICD‐10 diagnosis, was significantly lower in women identified at booking to be at increased risk of sPTB and referred to the PTBC, compared with those women not identified to be at increased risk of sPTB. These data support our hypothesis that women at increased risk of sPTB because of their past obstetric history, or other maternal factors, are less likely to suffer HG in their pregnancy.

These data are in keeping with the work of Vandraas et al., who identified an inverse association between HG and sPTB (<32 weeks) when analyzing the Medical Birth Registry of Norway.^12^ In contrast, previous studies show correlations between HG and an increased risk of PTB,^9^, ^10^, ^21^ which may be explained by their heterogeneous definitions of HG^12^ and their inclusion of all PTB, both iatrogenic and spontaneous. Protracted HG can be associated with fetal growth restriction leading to iatrogenic PTB.^22^, ^23^

The results of this study are in keeping with the concept that women who deliver spontaneously preterm have an altered hormonal response to pregnancy, which may in turn be associated with abnormal placentation. The known association between HG persisting into the second trimester and later placental dysfunction disorders, such as pre‐eclampsia and placental abruption,^24^ supports this hypothesis. Moreover, a recent meta‐analysis^6^ identified two studies showing an association between high serum progesterone concentration and HG.^7^, ^8^ Thus, although these studies were done in independent cohorts, it could be hypothesized that higher progesterone concentrations confer a lower risk of sPTB. The lack of association between progesterone concentration and HG described in the third study^25^ identified by Niemeijer et al. may be explained by their categorization of women's severity of nausea and vomiting according to patient‐completed questionnaires, rather than the more objective NICE standard definition for HG.^5^

Additional work is needed to explore the difference in admission rates for HG between women with risk factors for sPTB and those without these risk factors. A prospective study comparing serum or salivary progesterone levels and pregnancy outcomes in the two groups may be useful in this respect.

## 5 STRENGTHS AND LIMITATIONS

This analysis is limited by the sample sizes of the subgroups within the PTB HG group; these are insufficient to allow subgroup analysis, which prevents us from studying whether there is a link between HG and gestational age at delivery. Furthermore, complete outcome data are not available for non‐PTBC women not admitted with HG; it is therefore impossible to infer what proportion of the non‐PTBC cohort actually delivered spontaneously preterm. Although this limits the conclusions that can reasonably be drawn from this study, it does not compromise the observation that women with risk factors for sPTB are less likely to be admitted for HG than women without these risk factors.

Notably, the incidence of HG in the non‐PTBC cohort (3.33%) is higher than figures quoted elsewhere: 0.8%,^12^ or 0.5‐2%.^18^ This is likely to be because of the variability in coding definitions used in the studies to calculate HG incidence. Similarly, the rate of termination of pregnancy in our cohort was higher than the age‐standardized termination rate for England and Wales (1.59% in 2013)^26^ but is lower than that reported among women hospitalized with HG in a large multi‐center study (10.6%).^27^

We excluded women who had been treated with progesterone from the analysis on the grounds that this may have altered their risk of HG. The PTBC practice followed the RCOG statement at the time: to only prescribe progesterone prophylaxis for preterm birth within the confines of a clinical trial. The PTBC was recruiting to the OPPTIMUM randomized controlled trial.^20^ However, some women were referred having been prescribed progesterone by medical practitioners outside the PTBC. The small number of women who took prophylactic progesterone (n=18) therefore excluded those most at risk of sPTB. The decision to exclude these women may have introduced a degree of selection bias; however as the residual PTBC group was consequently composed of women with a lower risk of sPTB it is likely that the effect of this selection would be to reduce any difference in HG admissions between the two groups. We are therefore confident that the difference observed is not the result of selection bias.

Finally, although we excluded women with certain key individual risk factors for HG—including multiple pregnancy, presence of trophoblastic disease, and thyroid disease^19^—others were not identified in this study and thus, the following confounding factors were not considered: age; ethnicity; parity; sex of fetus; and family history of HG. Moreover, this analysis was not able to identify antenatal progesterone use in the non‐PTBC cohort. This necessitates some caution with the conclusions drawn.

## 6 CONCLUSION

In summary, this study found that hospital admission for HG in women at increased risk of sPTB was reduced compared with women with no identifiable risk factors for sPTB. This supports our hypothesis that HG and sPTB are clinical manifestations of opposite extremes of a biochemical spectrum, and suggests that further exploration of the pathogenesis of HG may facilitate our understanding of the mechanisms underlying sPTB.
